# IDPM: an online database for ion distribution in protein molecules

**DOI:** 10.1186/s12859-018-2110-9

**Published:** 2018-03-16

**Authors:** Xilun Xiang, Haiguang Liu

**Affiliations:** 1 Complex Systems Division, Beijing Computational Science Research Center, Beijing, 100193 China; 2 School of Software Engineering, University of Science and Technology China, Su Zhou, Jiangsu 215123 China

**Keywords:** Ion distribution, Protein, Statistical analysis, Database

## Abstract

**Background:**

Interactions between ions and proteins have been extensively studied, yet most of the studies focus on the ion binding site. The binding mechanism for many ion binding sites can be clearly described from high resolution structures. Although knowledge accumulated on a case-by-case basis is valuable, it is also important to study the ion-protein interaction statistically. From experimentally determined structures, it is possible to examine the ion distribution around each amino acid. Such distributions can reveal relation between ions and amino acids, so it is desirable to carry out a systematic survey of ‘ion-amino acid’ pairing interaction and share the information with a publicly available database.

**Results:**

The survey in the Protein Data Bank (PDB) revealed that approximately 40% of molecules records contain at least one ion. To reduce the bias resulted from protein redundancy, the statistics were extracted from a non-redundant dataset by excluding the proteins with similar sequences. Based on the structures of protein molecules and the location of ions, the statistical distributions of ions around each proteinogenic amino acid type were investigated and further summarized in a database. To systematically quantify the interactions between ions and each amino acid, the positions of ions were mapped to the coordinate system centered at each neighboring amino acid. It was found that the distribution of ions follows the expected rules governed by the physicochemical interactions in general. Large variations were observed, reflecting the preference in ‘ion-amino acid’ interactions. The analysis program is written in the Python programming language. The statistical results and program are available from the online database: ion distribution in protein molecules (IDPM) at http://liulab.csrc.ac.cn/idpm/.

**Conclusion:**

The spatial distribution of ions around amino acids is documented and analyzed. The statistics can be useful for identifying ion types for a given site in biomolecules, and can be potentially used in ion position prediction for given structures.

**Electronic supplementary material:**

The online version of this article (10.1186/s12859-018-2110-9) contains supplementary material, which is available to authorized users.

## Background

Ions are essential in biology, contributing to structural, catalytic, and regulatory aspects of molecules in cells [1, 2]. At the molecular level, ions take part in the structure, stability, and functions of biological molecules, such as DNA, RNA, and proteins. For example, ions often contribute to active sites of biological molecules, as in the case of the magnesium ions in chlorophyll of photosynthetic complexes [3], or the iron ions in the heme groups of hemoglobin [4]. Ions are involved in biochemical reactions through many enzymes, where they serve as co-factors that influence the reaction rates [5]. For example, manganese ions are found in the metal cluster in photosystem II [6, 7]. At cellular or larger scales, ions are responsible for signal transduction, maintenance of electrolyte balance and even the control of cell shapes and motions. A well-known example is the calcium ion, which is critical for transducing signals through the cytoplasm and into the nucleus to direct gene expressions [8, 9]. The signaling in the neurosystem is also heavily dependent on calcium ion flux through the neuron networks [10, 11].

The interplay between ions and biomolecules such as proteins and nucleic acids has been extensively studied [12]. It is known that certain ion binding sites are highly selective, and the tight binding is achieved by fine tuning the coordination geometry at the binding site in the nearest neighboring amino acids (first layer). The second layer around the binding sites are implemented by the interactions between amino acids through physicochemical interactions, such as coulombic interactions. Based on the accumulated knowledge, several databases have been constructed to allow interactive browsing into the ion binding site and examination of the details of ion coordination and geometry [13–15]. These databases have revealed rich information regarding how the ions are coordinated in each specific site by the neighboring amino acids. For example, MetalPDB extracts the metal ion binding sites from experimentally determined structures, and records the minimal functional sites (MFSs), thus facilitating the comparison of binding sites and the search for similar functional sites [15]. The MESPEUS database focuses on the ion coordination environment and the geometry of metal sites in proteins, revealing how ions with similar chemical properties can be discriminated by the finely tuned binding sites [14]. In this work, we provide an innovative approach to treat the same problem: instead of focusing on ions, we investigated where the ions are located relative to each neighboring amino acid. Unlike the MetalPDB or MESPEUS that describe the binding site in details, we shift the focus to the basic building blocks of proteins, the amino acids, and examine where ions are located relative to their neighboring amino acids. In addition to the metal ions, we included the analysis of the simple anions (F-, Cl-, Br-, and I-) from the 17th family of the periodic table.

The article is organized as follows: we first describe data acquisition and the details of analysis method; then the features of the ion database and major results are summarized; we demonstrate how statistical knowledge gained from data mining can be applied to predict the ionic type in a protein structure. The implication and potential applications of the statistical information are also discussed.

## Methods

### Protein structure dataset

The structures were downloaded from the protein data bank [16]; 51,687 out of 130,065 structures had at least one ion molecule. An ‘ion-amino acid’ pair is included in the analysis, if the distance between the ion and the geometry center (GC) of the amino acid is within 6 Å. The choice of cutoff at 6 Å is for the consideration that only the directly interacting ions for each amino acid should be included. There are size variations for amino acid side chains, we decided to choose this cutoff based on the main chain atom distances. From PDB files, the atomic coordinates of the ion and the associated amino acids for each ‘ion-amino acid’ pair were extracted for further analysis. Only the heavy atoms (non-hydrogen) were included in the analysis.

### Coordinate system definition

To analyze the spatial distribution of ions around each amino acid, we defined a local coordinate system for each amino acid, using the following procedure:

1. The origin of each local coordinate was set to be the geometry center of the amino acid;
2. The X-axis direction, $\widehat{x}$, was defined as the unit vector pointing from the origin to the backbone nitrogen atom;
3. The ***y***′ direction was defined as the vector from the origin pointing to the carboxyl carbon atom. The Y-axis can be calculated by removing the projection of ***y***′ on $\widehat{x}$:
  1 $$ \boldsymbol{y}={\boldsymbol{y}}^{\prime }-\left(\widehat{x}\bullet {\boldsymbol{y}}^{\prime}\right)\widehat{x} $$
  and then the unit vector for the Y-axis was obtained by normalization:
  2 $$ \widehat{y}=\boldsymbol{y}/\left|\boldsymbol{y}\right| $$
4. The Z-axis was defined as the direction perpendicular to x-y plane by cross product operation, i.e.,
  3 $$ \widehat{z}=\widehat{x}\times \widehat{y} $$

**Fig. 1:**
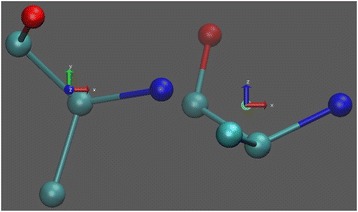
The coordinate system for alanine. The X-axis is defined by a unit vector pointing from center to the nitrogen atom; the Y-axis is the direction pointing to the carboxyl carbon atom and perpendicular to X-axis. The right-hand rule is applied to define the Z-axis based on the X-Y plane

The coordinate system for alanine at two orientations is shown in Fig. 1 to illustrate the axes.

For each ion around the amino acid, we defined ***p*** as the difference vector from the origin (i.e., the geometry center) to the ion position in the protein coordinate system, then the coordinates (*x*, *y*, *z*) around the amino acid can be represented as:

4 $$ \boldsymbol{p}=\left(\boldsymbol{p}\bullet \widehat{x}\right)\widehat{x}+\left(\boldsymbol{p}\bullet \widehat{y}\right)\widehat{y}+\left(\boldsymbol{p}\bullet \widehat{z}\right)\widehat{z}=x\widehat{x}+y\widehat{y}+z\widehat{z} $$

The Cartesian coordinates (*x*, *y*, *z*) were converted to the polar coordinates (*r*, *θ*, *φ*) by the following:

5 $$ r=\sqrt{x^2+{y}^2+{z}^2},\kern0.5em \theta ={\cos}^{-1}\frac{z}{r},\kern0.5em \varphi ={\tan}^{-1}\frac{y}{x} $$

Note that the coordinates (*x*, *y*, *z*) at the local coordinate system of each amino acid remain constant when the amino acid undergoes shifting or rotational transformations, if the conformational changes of the amino acid are ignored. Here, we consider each amino acid as a rigid building block for proteins to simplify the ion position mapping.

After establishing the coordinate system, we calculated the coordinates of the ions around each amino acid. By going through all structures in the database, a distribution function for each ion around each type of amino acid was obtained.

### Ions and side chain group

We defined a quantity to measure the preference of the ion towards side chains as the following:

6 $$ R=\frac{N_s}{N_s+{N}_c} $$

where *N*_*c*_ and *N*_*s*_ are the number of ions within *r*_*a*_ of the geometry centers of the amino acid backbone and side chain, respectively. The new cutoff *r*_*a*_ in this analysis was defined as *r*_*a*_ = 6 − max(*r*_*c*_, *r*_*s*_), where *r*_*c*_ and *r*_*s*_ is the distance from the center of backbone atoms or from the center of side chain atoms to the geometry center of the whole amino acid. This quantity was not defined for glycine, because the side chain is composed of a single hydrogen atom. The larger the *R* values, the stronger the attraction between the side chain and the paired ion.

### Hierarchical clustering of amino acids based on the spatial distribution of ions

The amino acids with similar physicochemical properties tend to attract or fix ions that are alike, so we used the ion distribution functions surrounding amino acids as features to classify the 20 amino acids. The correlation coefficients between spatial distribution functions were computed using an Fast Fourier Transform (FFT)-based fast rotation function [17]. The 20 × 20 similarity matrix was then used for classification using a hierarchical algorithm with complete linkage clustering, i.e., the distance between the two clusters were defined as the maximum distance between any pairs of elements in two clusters.

### Webserver setup

The IDBM webserver was designed to allow users to visualize ion distributions interactively using the web interface. The distributions are mainly displayed using the Jmol plugin [18]. The animations are rendered using the VMD program to show the rotating pictures of the distributions in 3D space [19]. The webpages were developed using the bootstrap framework (https://getbootstrap.com/), the main contents include motivation of this study, basic information about ions and amino acids, data analysis methodology, and statistical results.

### Ionic type prediction

The preference in ‘ion-amino acid’ contacts can be utilized to make predictions about ionic types for specific positions in given structures. In other words, if an ion (with an unknown type) exists in a protein, the knowledge from statistical analysis can be used to evaluate the probability of observing each ion type. The probability is inferred from the joint probability of co-existing ‘ion-amino acid’ pairs. The physicochemical environment at any position was defined by the neighboring amino acids, and the corresponding probability of ionic type, *p*(**r**, ion) at position ***r*** was expressed as:

7 $$ p\left(\boldsymbol{r}, ion\right)={\prod}_{\left\{ aa\right\}}\frac{p_{\left( aa, ion\right)}}{p(aa)p(ion)} $$

where {*aa*} is the set of neighboring amino acids, *p*(*aa*) and *p*(*ion*) are the probabilities of amino acid type *aa*, and ionic type *ion*; *p*(*aa*, *ion*) is the probability of observing the ‘ion-amino acid’ pair. All the probabilities are derived from statistics, only the set of neighboring amino acids are set by the structure and the given position ***r***.

This above formula (Eq. 7) does not fully utilize the *spatial* distributions of ions, because the ions are not uniformly distributed around the amino acids. Therefore, we implemented a more advanced algorithm that divides the distributions into angular regions based on the angles in the polar coordinates. Then, Eq. (7) becomes

8 $$ p\left(\boldsymbol{r}, ion\right)={\prod}_{\left\{ aa\right\}}\frac{p\left( aa,\theta, \varphi \right)}{p_{(aa)}{p}_{(ion)}} $$

Here, the (*θ*, *φ*) defines the location of the ion relative to the center of amino acid using Eq. (5), the other symbols are the same as in Eq. (7). To simplify the calculation, we divided the sphere into eight sections, corresponding to the octants based on the signs of Cartesian coordinates. All the distribution parameters used in Eqs. (7) and (8) are derived from the minimal non-redundant structure dataset.

## Results

### Abundance of ions observed in the structures of biomolecules

**Fig. 2:**
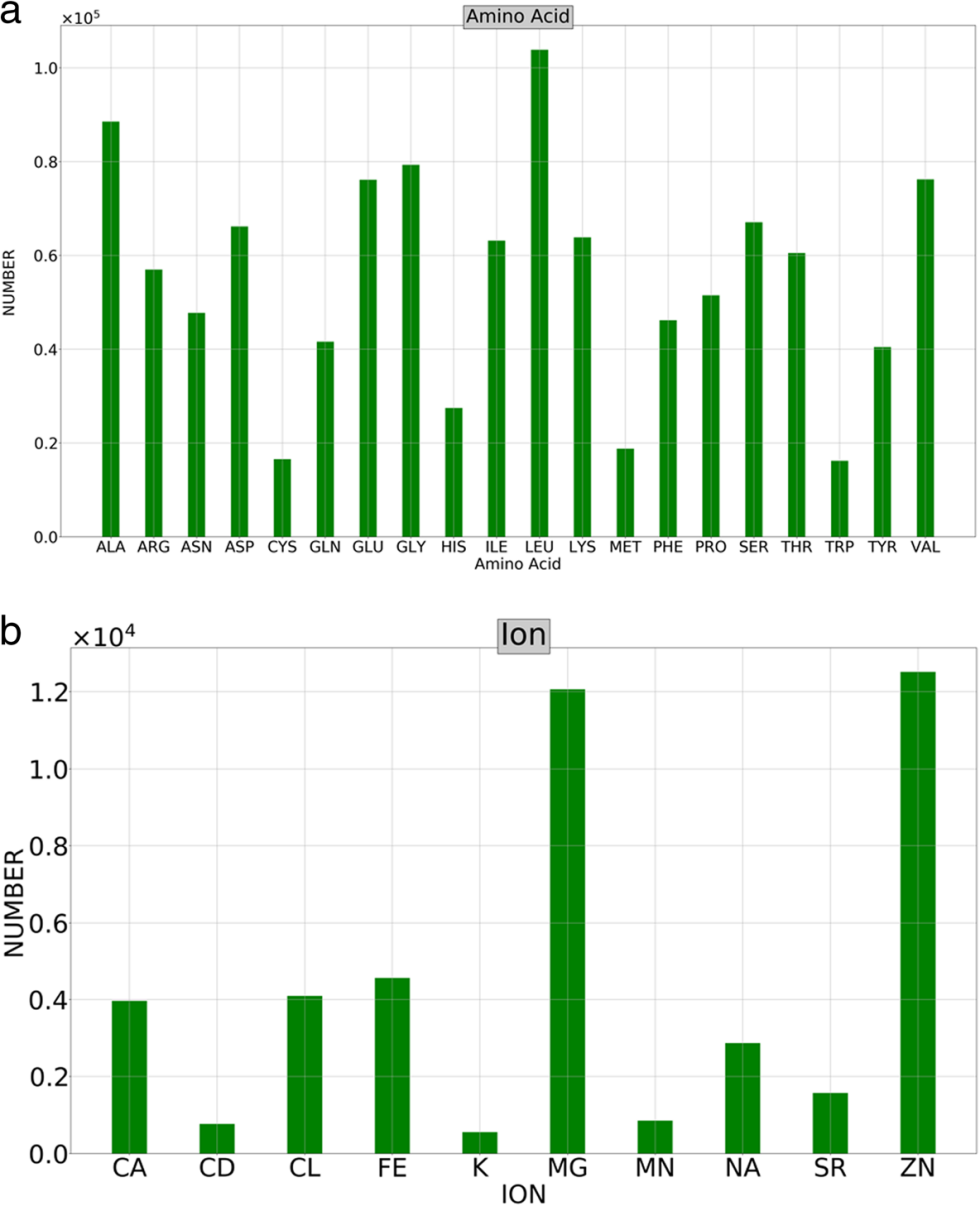
Abundance of amino acids and the most abundant ions observed near amino acids. **a** The number of amino acids from the 51,687 structures. **b** The number of 10 most abundant ions near amino acids

The protein data bank included 130,065 structures of biological molecules as of June 2017, out of which 51,687 structures of protein or nucleic acid contain at least one ion, corresponding to 39.7% of the whole database. In this study, we focus on the ion distributions around amino acids in protein structures. Furthermore, to avoid the bias in statistics due to redundant structures of similar protein molecules, we used the minimal non-redundant dataset in the statistical analysis. The number of amino acids varies significantly. Leucine, alanine, and glycine were the three most abundant amino acids, while the least observed amino acids were tryptophan, cysteine, and methionine (Fig. 2a). This trend is in agreement with the statistics from proteomics data [20]. The number of observed ions also have large variations. In Fig. 2b, the 10 most populated ions in the molecules deposited in the protein data bank were Ca^2+^, Cd^2+^, Cl^−^, Cu^2+^, Fe^2+^/Fe^3+^, K^+^, Mg^2+^, Mn^2+^, Na^+^, and Zn^2+^, which are essential elements in the human body and other living things [2]. For ions in nucleic acids, Mg^2+^ was much more abundant than other ions (data not shown, available from the online database). This is consistent with the fact that the magnesium ion is critical to the structure and function of nucleic acids [21].

### Contact preference of ions with amino acids

**Fig. 3:**
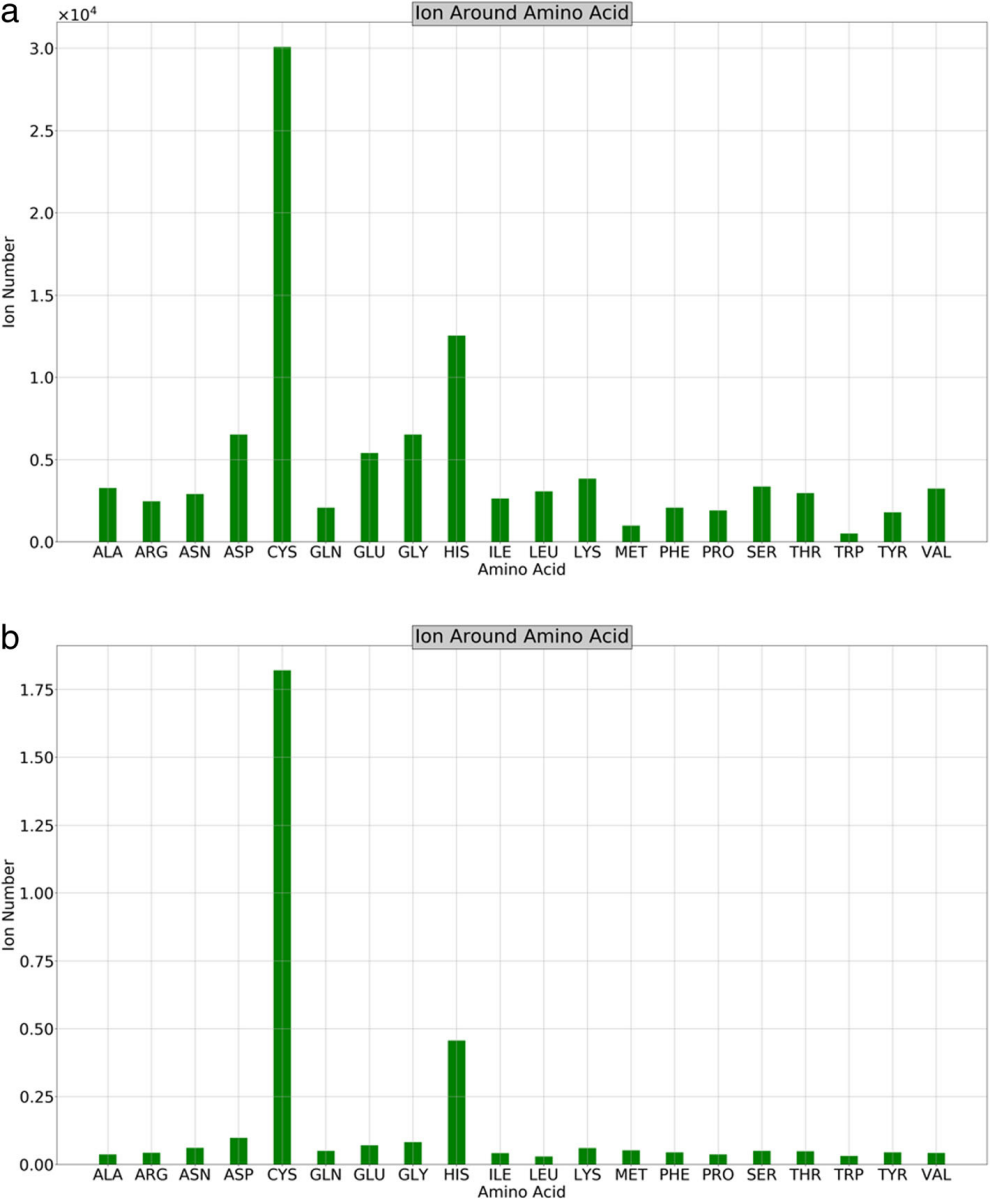
Number of ions observed near each amino acid. **a** Total number of ions observed near each amino acid; **b** the average number of ions for each amino acid, obtained by dividing the number of the corresponding amino acid from (**a**)

The number of neighboring ions for each amino acid varies largely, as shown in Fig. 3. For example, the number of ions found near cysteine was the largest among amino acids, followed by aspartic acid and histidine. It is worth pointing out that the data shown in Fig. 3a are the actual numbers of observed ions in the structure database, strongly correlated to the abundance of amino acids (see Fig. 2a). To remove the bias due to the different abundance of amino acids, we computed the average number of ions found near each amino acid (Fig. 3b). This trend illustrated in Fig. 3b is in accordance with the physicochemical properties of amino acids. For example, cysteine is well known for its capability of ion fixation, especially the largely abundant Zn^2+^ ions [22, 23]. The negatively charged amino acids have stronger attractive force to metal ions, yielding large ion populations around aspartic and glutamic acids. The hydrophobic amino acids are mostly buried inside protein cores, making the chance of observing ion molecules near these amino acids much smaller, which is consistent with the results shown in Fig. 3.

**Fig. 4:**
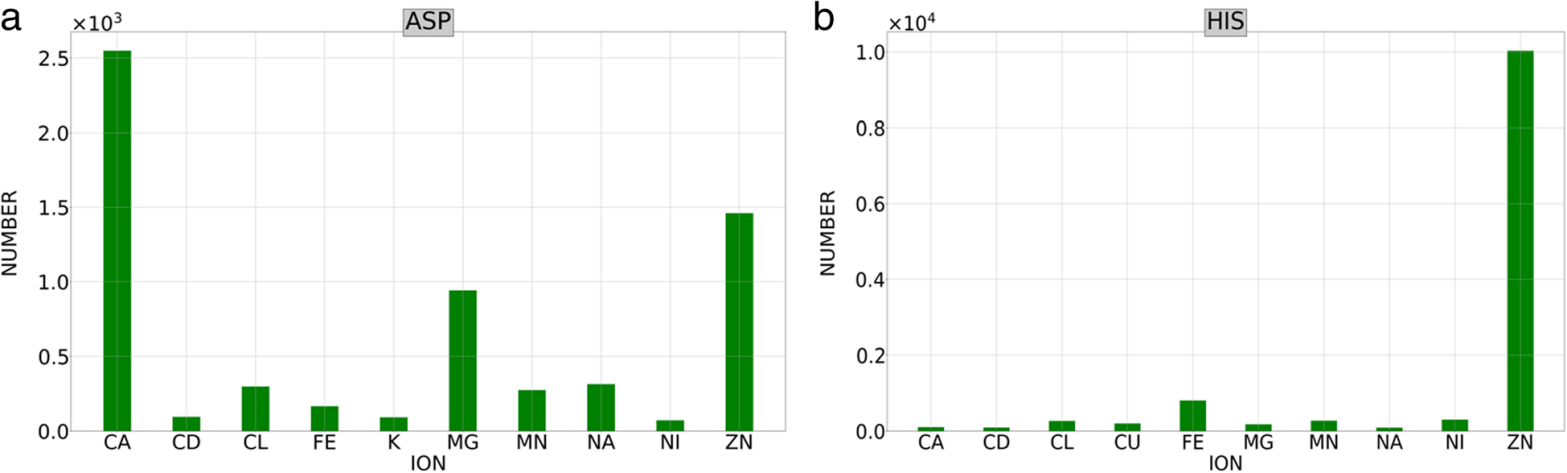
The population of ion types surrounding amino acids. Two representative cases are shown: **a** The ions within 6 Å of geometric center of aspartic acid; **b** the ions within 6 Å of geometric center of histidine

The type of ions neighboring each amino acid varies largely as well. For instance, the most abundant ions observed next to aspartic acid and histidine are shown in Fig. 4. Ca^2+^ was the most populated ion around the aspartic acid, while histidine was surrounded mostly by Zn^2+^. These observations verify that the aliphatic amino acid such as aspartic acid can be stabilized by complexation with Ca^2+^ ions. The crowding of Zn^2+^ ions near cysteine or histidine can be attributed largely to the DNA binding proteins, such as zinc-finger proteins that utilize CYS/HIS to coordinate the binding to Zn^2+^ ions, and Zn^2+^ in turn stabilizes the protein domains [23].

**Fig. 5:**
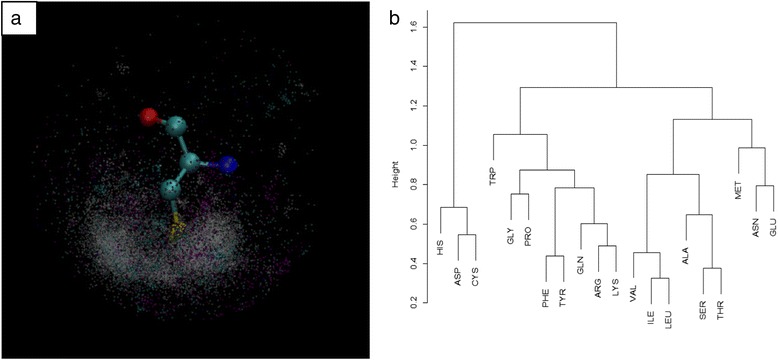
Inhomogeneous distribution of ions. **a** The ion distribution around cysteine. The zinc ions interact with the sulfur group closely, so they form a tight cluster (white dots), while other ions also exhibit strong preference towards the side chain. **b** The dendrogram of the hierarchical clustering results using ion distribution is as features of amino acids

The statistics indicate that the ions are not uniformly distributed around each amino acid; instead, they have strong positional preference relative to the amino acid. This is especially true for the amino acids with active or charged groups. For example, the distribution of Zn^2+^ around cysteine was one of the most extreme cases, with almost all Zn^2+^ ions located next to the sulfur atom on the side chain (Fig. 5a). As a comparison, the distribution of Zn^2+^ ions around alanine and lysine did not display strong preference on locations, relatively speaking. The positively charged side chain of lysine has repulsive effects to keep the Zn^2+^ ion some distance away. The distribution around alanine did not exhibit location preference, because the hydrophobic side chain generates weak electrostatic interactions except steric repulsion due to the short distance van der Waal interactions.

In general, the number of ions surrounding each amino acid had the following trend, in agreement with the strength of ion attraction/fixation: non-popar amino acids < polar amino acid with positive charge ~ polar amino acid (without net charge) < polar amino acid with negative charge (See Table 1). The observed trend is consistent with the rules based on physical principles: polar amino acids, especially the ones that carry net negative charges in cellular conditions, provide favorable environments for metal ions; and the amino acids with hydrophobic side chains are more likely to be buried in the hydrophobic core, which is unfavorable for charged ions. Most ions in the proteins are metal ions with positive charges, preferring the amino acids with negative charges.

**Table 1:** The number of ‘ion-amino acid’ pairs. The amino acids are grouped based on side chain properties

	Non-polar amino acid	Polar amino acid without charge	Amino acid with positive charge	Amino acid with negative charge
Number of observed pairs of ion-amino acid	ALA: 3284	ASN: 2318	ARG: 2478	ASP: 6541
	ILE: 2639	CYS: 30081	HIS: 12547	GLU: 5416
	LEU: 3074	GLN: 2082	LYS: 3854	
	MET: 986	GLY: 6543		
	PHE: 2081	SER: 3372		
	PRO: 1912	THR: 2973		
	TRP: 514	TYR: 1803		
	VAL: 3250			
Average	2218	7110	6293	5979

### Spatial distributions of ions around amino acids

As mentioned previously, the distributions of ions around each amino acid had large variations, depending on the properties of the side chains. For example, a subset of the ions around cysteine are shown in Fig. 5a, with cysteine represented using ball-stick mode, and the ion positions shown using point clouds that are colored based on elements. Most ions are concentrated near the sulfur atom, and the upper half sphere contains significantly fewer ions. To quantify the tendency of ions towards the side chains, we divided the ions into two groups depending on whether the ions are closer to the center of either the main chain or the side chain, with the procedure described in Method section (see Eq. 6). The percentage of ions that are closer to the side chain was calculated for each amino acid (Table 2). The amino acids with negatively charged side chains had stronger attractions to the ions (mostly carrying positive charges).

**Table 2:** The percentage of ions that are closer to the side chain for each amino acid. The cysteine, histidine, and negatively charged amino acids (aspartic acid and glutamic acid) have higher tendency towards side chains (in boldface)

	Non-polar amino acid	Polar amino acid without charge	Amino acid with positive charge	Amino acid with negative charge
	ALA: 57.5%	ASN: 67.1%	ARG: 68.2%	**ASP: 81.7%**
	ILE: 14.3%	**CYS: 94.5%**	**HIS: 98.0%**	**GLU: 92.1%**
	LEU 37.7%	GLN: 59.2%	LYS: 47.0%	
	MET: 66.4%	SER: 55.6%		
	PHE: 55.0%	THR: 57.2%		
	PRO: 44.7%	TYR: 11.1%		
	TRP: 0.0%			
	VAL: 56.0%			
Average	41.4%	57.5%	71.1%	86.9%

### Classification of amino acids based on ion distributions

The ion distribution around each amino acid can be used as a feature to describe the properties of the corresponding amino acid. The correlations between distribution functions were used to measure the similarity of amino acids. The resulting 20 × 20 similarity matrix was used to cluster the amino acids. The dendrogram of the hierarchical clustering result is shown in Fig. 5b. Three amino acids, aspartic acid, cysteine and histidine, were clustered into a sub-group, reflecting highly polarized distributions of ions around these amino acids. The nonpolar amino acids were in general clustered into another sub-group, as the ions are more evenly distributed around them. This clustering result also indicates that the ion distributions are mainly determined by the properties of each amino acid, especially the electrostatic interactions.

### Application of ion distributions in ion type prediction in proteins

**Fig. 6:**
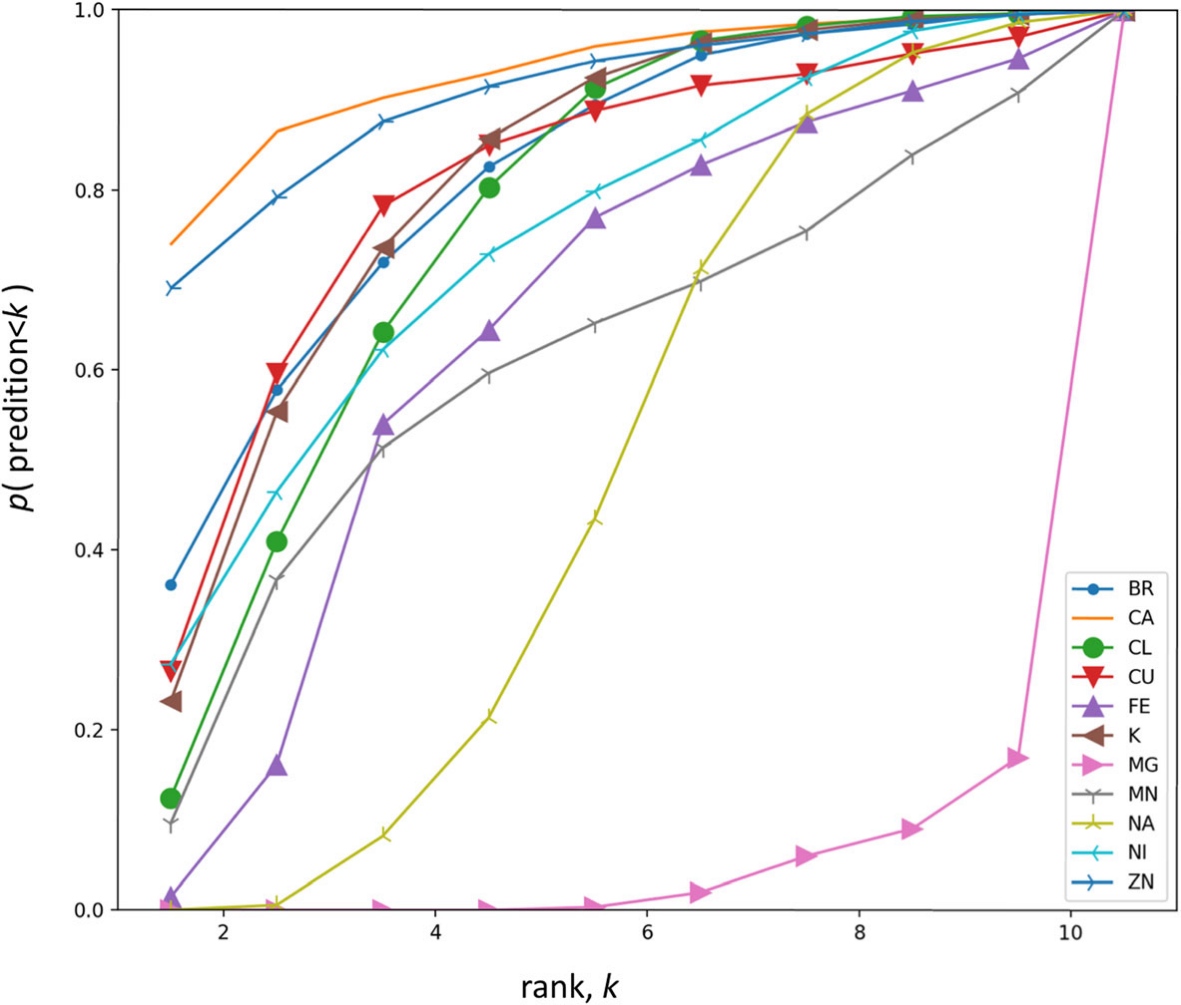
The cumulative probability distribution of the predicted ranks. Larger fractions at smaller ranks indicate better predictions

The ion binding site is well defined by the surrounding amino acid if the site is specific to the ion types. Using the statistics obtained from the PDB, we developed an algorithm to predict the ion type for given positions in any protein molecule. To demonstrate the performance at prediction, we randomly selected 1000 binding sites (except for Ni^2+^ ion, which has only 919 structures) from PDB models for each of the 11 most abundant ions, and blindly predicted the ionic type for each site. This testing dataset is composed of structures from the protein databank excluding the ones that are used for the statistics. The power of prediction was assessed by examining the ranking of each particular ionic type. Based on the probability computed using statistical information, the averaged ranking for each ion in the 1000 binding sites are summarized in Table 3. Smaller ranks correspond to better prediction accuracy (An average rank of 1 means the predictions are 100% correct) for that ion, reflecting higher binding specificities. The predicted rank distribution is computed for each ion. The cumulative probability is shown in Fig. 6 for the tested ions. We found that Ca^2+^ and Zn^2+^ had the most specific binding sites defined by the surrounding amino acid (average rank < 2), while the binding sites for alkali metals were not very specific based on the statistics and the prediction results. It is worth pointing out that ion channels for K^+^ or Na^+^ can be strictly selective, which is not contrary to our observations. Nonetheless, the averaged ranks are mostly below 6 (except for Mg^2+^), which is better than random selection (the expected rank is 6 for 11 elements). The ion abundance is not strongly correlated with the prediction power for the corresponding ion type.

**Table 3:** The averaged ranking based on predictions. We tested the performance of the algorithm by using the essential ions. The test dataset is composed of the structures that are not used in the statistics of ion distributions. The averaged ranks and their standard deviations are summarized. The two best predicted ionic types (Ca^2+^ and Zn^2+^) and the worst case (Mg^2+^) are highlighted in boldface

Ion	Average rank	Standard deviation
Br^−^	2.78	1.93
**Ca** ^**2+**^	**1.70**	**1.58**
Cl^−^	3.16	1.66
Cu^2+^	2.88	2.23
Fe^2+/3+^	4.26	2.16
K^+^	2.86	1.83
**Mg** ^**2+**^	**10.24**	**1.21**
Mn^2+^	4.59	3.03
Na^+^	5.73	1.62
Ni^2+^	3.37	2.31
**Zn** ^**2+**^	**1.81**	**1.72**

### The IDPM database and its features

The ion distribution in protein molecules, **IDPM database**, is designed to visualize the ion distributions and to provide a summary on quantitative analysis of distributions. The data described in the results section are fully available for interactive online visualization and downloads.

In the *main page*, the distributions of ions are shown in two formats: one figure is rendered using VMD, showing all ions around cysteine; and the other figure is Mg^2+^ around the nucleic acid adenine using the Jmol plugin that allows interactive examination of the distributions.

The *introduction page* provides the background of the study.

The *method* page describes how the datasets are obtained and analyzed, as well as the mathematical description of quantifications.

**Fig. 7:**
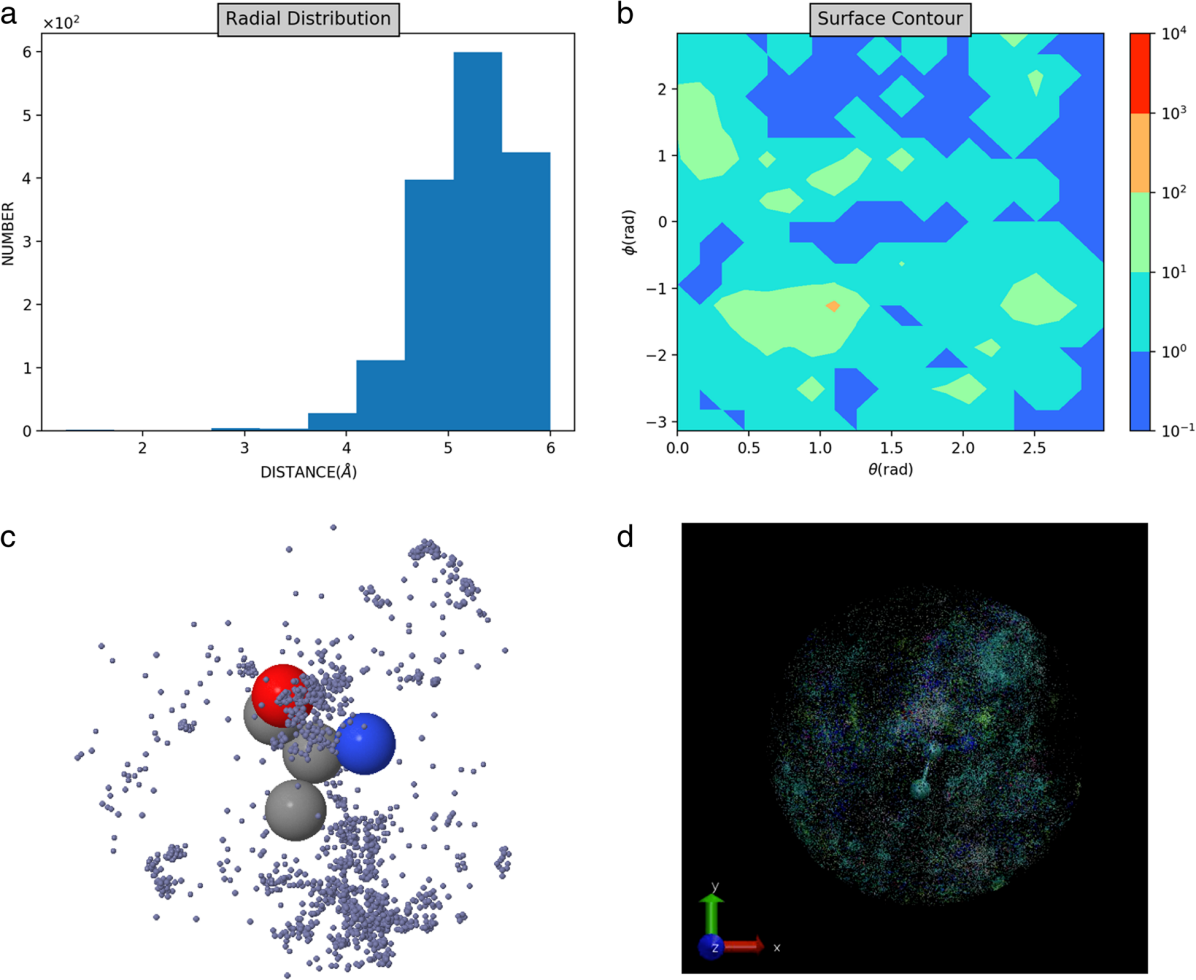
Ion distribution around alanine. **a** Radial distribution of zinc ion around alanine. **b** Angular distribution of zinc ion around alanine. **c** A snapshot of 3D distribution of all ions around alanine. **d** A snapshot of 3D distribution of zinc ion around alanine visualized using Jmol plugin in the webserver


The *amino acid page* summarizes ion distributions and key observations. Users can find the abundance of ions and amino acids, and examine the distribution of each specific ion type around each amino acid. The raw data and the individual distribution for each ‘ion-amino acid’ pair are also available for downloading in the dynamically generated pages. This raw data is saved in the format of PDB, such that the distributions can be easily visualized using all main stream programs. The entries of atom name, chain ID, and B-factor are used to save the PDB ID of the source structure, the chain ID in the source structure, and the distance to center of the corresponding amino acid. When a mouse cursor is moved to the ion, the PDB ID and chain ID information can be displayed. A summary for ions around alanine is shown in Fig. 7, including the radial distribution, angular distribution, a snapshot of 3D interactive distribution represented using Jmol plugin, and a snapshot of 3D distribution animation rendered using VMD (see Additional file 1 and Additional files 2, 3 and 4 for the videos).

Additional file 2:Ion distribution around aspartic acid. The 3D ion distribution around aspartic acid shown in video rendered using VMD. (MP4 2605 kb)
Additional file 3:Ion distribution around cysteine. The 3D ion distribution around cysteine shown in video rendered using VMD. (MP4 2609 kb)
Additional file 4:Ion distribution around histidine. The 3D ion distribution around histidine shown in video rendered using VMD. (MP4 2608 kb)


The *nucleic acid page* shows the ion distributions around nucleic acids. Because the number of structures with ions associated to nucleic acids is small (about 1300 for RNA and 1700 for DNA molecules), the results extracted from such dataset are lack of statistical significance. Therefore, we only present the preliminary results in the original form, without thorough discussion as for the case of amino acids.

The *ionic type prediction page* is an application of the IDPM. The users can upload a protein structure and specify a location in the molecule, then the program will predict the possible ion types based on the neighboring amino acids and the associated statistical information.

The data with all the figures and analysis source codes (including a documentation file) can be obtained from the *download page*.

## Discussion

### Convergence of statistics

Over 130,000 structures have been deposited in the Protein Data Bank, and it is not unusual for the structures of the same protein to be deposited multiple times; and the proteins with similar sequences (which often fold into similar structures) add more complexity to the choice of representative structure dataset. The statistics presented in this work were carried out with this redundancy under consideration. To test the consistency of the statistics from the database and related subsets using the same protocol, we analyzed the whole PDB and four subsets at various non-redundant levels, obtained from the NCBI VAST (the vector alignment search tool) server (see Table 4 for the detailed information). The protein structures with ions make up about 40% of each subset, in spite of different redundancy levels. In another subset composed with only structures with resolution better than 2.5 Å, about 47% of which contain ions. This higher percentage compared to other datasets is likely attributed to the fact that high resolution structures reveals the positions of ions better than the structures with lower resolutions. Considering that structures with high resolutions provide higher confidence, the analysis was also carried out for this subset of structures.

**Table 4:** Basic information about the structure dataset and non-redundant subsets. The non-redundant datasets contain only protein structures, the high resolution and whole PDB datasets have both structures of protein and nucleic acid. In the analysis for ion distribution around amino acids, the nucleic acid structures are not included

DataSet	# structures	# structures with ions	Ratio
*p*-value 10E-7	14,647	4938	0.34
*p*-value 10E-40	25,546	9228	0.36
*p*-value 10E-80	36,455	12,685	0.35
Non-identical	70,902	26,717	0.38
High resolution (< 2.5 Å)	56,711	26,417	0.47
Whole PDB	130,065	51,687	0.40

**Fig. 8:**
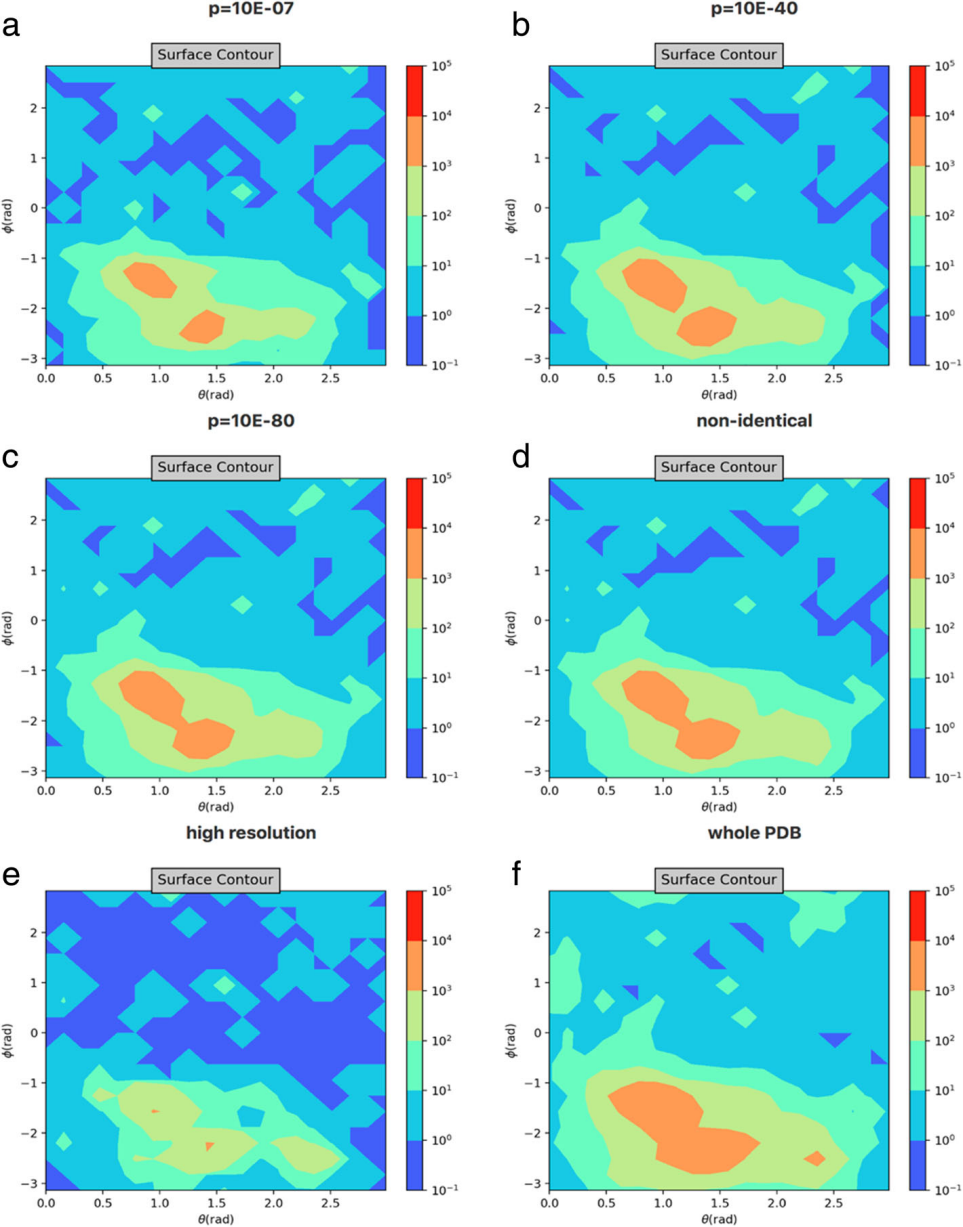
The 2D angular distribution of zinc ions around cysteine for different datasets. **a** The results for minimal dataset used in this study with VAST *p*-value = 10E-7. **b** and **c** are the results for *p*-values of 10E-40 and 10E-80 respectively. **d** The results for non-identical dataset. **e** The results for high resolution dataset. **f** The results for the whole PDB dataset

The statistics we reported here are highly consistent by cross comparing ion distributions obtained from the six datasets described previously. For instance, the angular distributions of the zinc ion around cysteine are shown in Fig. 8 to illustrate the consistency. In spite of small variations in the angular distributions, the overall trends are highly similar, with a dominant population of zinc ions distributed at the lower left region. The statistics obtained from each dataset are consistent with that from other datasets. The presented data here and in the IDPM webserver are from the minimal non-redundant dataset. The results from the other datasets are also available for download and comparison.

As the number of experimentally determined protein structure keeps increasing, the statistics presented in this work should be updated regularly. The analysis program is written in Python programming language with utilizations of built-in libraries for intensive data process sections. On a computer with 3GHz Intel Xeon E5, it takes about 20 min to complete the analysis, including the generation of all statistics and figures using a single processor. Therefore, it is straightforward to update the database with any new dataset to keep up with the Protein Data Bank or the NCBI VAST datasets.

### The ion location prediction

The statistical distribution of ions in biomolecules can be used to predict the probable ion types for a given position within protein molecules, as demonstrated in the results section. Another possible application is to predict the location of ions in proteins. As each ion has its preferred locations relative to each amino acid, these preferences can measure the relative binding strength, and at the least, provide qualitative rankings. We are working on developing sophisticated scoring functions derived from the statistical distributions obtained from the protein database. This is useful for ion binding site analysis and protein structure refinement at the presence of ions.

It is worthwhile to point out that the current statistics based on structures are limited to the stably bound ions. The transient interactions between ions and proteins are often not determined using crystallography or nuclear magnetic resonance (NMR) methods, therefore, they are underrepresented in the current database. Furthermore, although most of the ions found in protein molecules have biological functions, the results from the PDB structures can be mixed with ions that are used for phasing (Br^−^ for instance can be used for anomalous diffraction phasing). Nonetheless, these heavy atoms must have stable binding to specific sites to facilitate electron density map reconstructions. The neighboring amino acids of such ions also form very well coordinated electronic environments, so the analysis presented in this study is also valid.

## Conclusions

We analyzed the structures deposited in the Protein Data Bank and obtained an overall understanding of ion distributions around amino acids. The statistical analysis reveals the ‘ion-amino acid’ interaction follows the physicochemical rules in general, and the ion abundance and distribution depend on the properties of amino acid side chains. The spatial distribution of ions can be applied to calculate the probability of occurrence for each ion at a given position in a protein molecule. All the data and software are available at http://liulab.csrc.ac.cn/idpm/.

## Additional files

Additional file 1: Supplementary materials. Additional information about the IDPM database. (DOCX 39 kb)

## Abbreviations

IDPM: Ion distribution in protein molecules
NCBI: National Center for Biotechnology Information
PDB: Protein data bank
VAST: The vector alignment search tool
