# Supplementary material for: IDPM: an online database for ion distribution in protein molecules
Source: BMC Bioinformatics. 2018 Mar 16;19:102. doi: 10.1186/s12859-018-2110-9 (PMC5857119; doi:10.1186/s12859-018-2110-9)
Supplement: Supplementary file 4 — Supplementary materials. Additional information about the IDPM database. (DOCX 39 kb) [file 12859_2018_2110_MOESM1_ESM.docx]

**IDPM: an online database for ion distribution in protein molecules**

Haiguang Liu, Xilun Xiang

All the ion distributions are available for download at <http://liulab.csrc.ac.cn/idpm/>.

Here, we provide three examples to demonstrate the highly polarize distributions in three amino acids:

aspartic acid, cysteine and histidine.

The videos were rendered using VMD.

ASP.mp4

CYS.mp4

HIS.mp4
